# ‘Kindness and empathy beyond all else’: Challenges to professional identities of Higher Education teachers during COVID-19 times

**DOI:** 10.1007/s13384-022-00552-1

**Published:** 2022-08-04

**Authors:** Melissa Cain, Chris Campbell, Kathryn Coleman

**Affiliations:** 1 School of Education, Australian Catholic University, 1100 Nudgee Rd, Banyo, QLD 4014 Australia; 2 Division of Learning and Teaching, Charles Sturt University, 386 Elizabeth Mitchell Drive, Albury, NSW 2640 Australia; 3 Melbourne Graduate School of Education, University of Melbourne, Grattan St, Parkville, VIC 3010 Australia

**Keywords:** Higher education, COVID-19, Teaching and learning, Technologies, Educational inequity

## Abstract

COVID-19 has continued to effect higher education globally in significant ways. During 2020, many institutions shifted learning online overnight as the sector closed its doors and opened new sites for remote teaching. This article reports on an international study [Phillips et al., 2021] that sought to capture how cross-sectoral teachers experienced these emergency changes during the first months of restrictions. The data, analysed using narrative identity theory, revealed concerns that fall into two broad categories: technologies and relationships. Significantly, it was not a loss of content delivery or changes to assessment that prompted the greatest anxiety for our colleagues, but that they held significant concerns about their students’ mental health; inequities of access to a range of services including technological; and challenges connecting emotionally with their students at a distance. The results provide actionable strategies for higher education institutions to apply in future emergencies where remote teaching is necessary.

## Introduction

This research forms part of a larger cross-sectoral study investigating teaching and learning during COVID-19 (Phillips et al. 2021). As academics pivoting our teaching and research during the pandemic, we became interested in the rich data set of higher education (HE) stories as we began to analyse. Exploring the 105 respondents in the HE/tertiary sector we were able to focus on the impacts on the academy and its ability to pivot to remote emergency teaching. We discovered that the acute change to offering HE courses online when COVID-19 restrictions were introduced impacted learning and teaching in multiple ways. The logical suggestion that learning and teaching could simply be ‘moved online’ did not easily account for issues concerning equity of access and indeed found in the data stories, was seen to magnify existing sociocultural inequities (Cain & Phillips, 2021; Phillips et al. 2021). Additionally, for educators who primarily interact with their students face-to-face, their professional identities and agency were challenged during this time.

Naylor and Nyanjom (2020) suggest that teaching “is deeply connected to one’s beliefs, values, commitments and relationships with students” (p. 1). The relationship that teachers have with their profession evolves with reference to the educational contexts that teachers find themselves in, their personal agency in these contexts, and changing professional experiences (Murray et al., 2011). Teachers in HE contexts in particular, affiliate themselves with certain professional practices and academic communities, whilst choosing to reject others (Yuan, 2016). They hone their professional identities over time, defining their personal approach to the craft of disciplinary teaching through successful encounters with learning. Considering the complex social and cognitive processes involved, teaching is, therefore, inherently personal, and emotionally laborious (Yin, 2015).

Teachers often speak of the rich connections they make with their students that cannot be adequately described or measured. These connections rely heavily on emotional intelligence and often lead to a higher sense of professional accomplishment (Platisidou, 2010). This may, for example, include experiencing emotional contagion, which is the transference of emotional states. That is, if students view their teachers as positive and complimentary, they may mimic their teacher’s behaviours, using nonverbal reactions. This type of feedback serves to predict how a teacher perceives their teaching within the classroom. These positive interactions contribute to building a teacher’s self-confidence and self-efficacy and confirm that their teaching style is effective and appreciated. Significantly for this pandemic study, when HE teachers experience positive emotions, they feel “energised and motivated to try out new technologies and ways of engaging with students” (Naylor & Nyanjom, 2020, p. 3).

In this article, we explore how teachers in HE contexts were forced to examine their conceptions of teaching and professional identities in response to using digital technologies as their primary source of connecting with students during the pandemic. The aim was to answer the overarching research question: ‘in what ways did the immediacy of the shift to online learning during COVID-19 times impact teachers’ identities?’ To provide context to the data and analysis of the larger study (Phillips et al. 2021), a review of the literature covering the relevant themes that emerged in our data analysis is presented here, including, responses to moving learning online, learning new technologies and applications, and teacher and student well-being.

## Literature review

### Responses to moving learning online

Globally there has been a steady increase in online delivery in HE (Morris et al., 2020) partly due to physical campuses constrained in their ability to grow, but also due to increased equity of access to education for a wider range of learners (Dodd, 2021; Stone et al., 2016). At the same time, there has been a purposeful move towards student-centred learning (Ryan et al., 2022), and a focus on active and problem-based learning strategies (Chernikova et al., 2020; Lee et al., 2014). Teachers often view themselves as proficient in either face-to-face or online delivery as a component of their professional identity (El-Soussi, 2022). Knowing that the consequential connection between teachers’ epistemic beliefs and values (Knorr Cetina, 2007; Norton et al., 2005) and their relationship to their students, a change in pedagogy and mode of content delivery can “disrupt these deep and personal connections giving rise to an emotional response” (Naylor & Nyanjom, 2020, p. 1). Making a sudden change from face-to-face to online delivery has the potential to cause experienced educators to feel deskilled, vulnerable, or isolated (Downing & Dyment, 2013). Kim and Asbury’s (2020) research with teachers from state schools in the UK notes that in reacting to multiple stressors resulting from teaching during COVID-19 restrictions, teachers used two types of coping strategies: problem-focused (“intended to alter the source of the stress”), such as working to increase proficiency and skills, and emotion-focused strategies (“intended to alter the emotional experience of stress”) such as seeking emotional support from peers and education communities (p. 3).

Naylor and Nyanjom’s (2020) timely research with 20 educators with varying experience in online teaching at an Australian university identified four HE teacher orientations towards online teaching and learning: futuristic educators; ambivalent educators; disillusioned educators; and cautious educators (p. 7). Those labelled futuristic educators revealed positive emotions towards online learning and perceived they received a high level of institution support. These educators applied a constructivist and developmental approach to understand the change from their students’ perspectives. Overall, they described feeling “positive, enthusiastic, and motivated towards the changing landscape of teaching” (p. 8). What is important to note here is that this confidence was associated with reciprocal long-term planning and a sense of teamwork towards shared goals. Contrastingly, those described as ambivalent educators accepted the move to online learning somewhat pragmatically. They were willing to compromise but received less institutional support. As such, these educators undertook the journey predominantly by themselves, figuring it out as they went. Whilst disillusioned educators had strong and comprehensive institutional support and anticipated the success of online learning, they were, at the same time, frustrated with technological limits of the learning management system and the resulting lack of innovation in the online space. Educators labelled ‘cautious’ in this study, mainly experienced negative emotions towards online learning, feeling that the new educational landscape was beyond their control, and that online learning was primarily a passive form of teaching. They worried about how the development of practical skills and delivery of formative feedback would be impacted, and how they would motivate and engage students if not in the same physical space. Naylor and Nyanjom (2020) note that the educators in this group described feeling “disempowered, resentful and undervalued” (p. 11).

### Learning new technologies and applications

One of the major areas of concern noted in the literature that discusses teachers’ reactions in moving to online teaching and learning during COVID-19 restrictions, was the feeling of being unprepared for teaching solely using digital technologies. This evoked an initial emotional reaction for many. In Watermeyer and colleagues’ (2020) research with 1148 university educators in the UK, respondents described feeling ill-prepared, lacking confidence, and lacking sufficient institutional support. In reference to the abrupt nature of moving online (with many teachers having only two days’ notice) one respondent lamented “I would like to engage in more training to become a more tech-savvy lecturer, but with no time to prepare, play, explore the technologies available, at the moment I am merely stressed out of my wits, as are my students” (p. 11). Respondents also expressed their reluctance or unwillingness to learn new technologies and skills; seemingly resigned to a negative outcome with one person stating “I have no real training in online course design. I have no interest in teaching like this anyway, and there is no possible way I am going to invest in completely re-doing this course on the fly” (p. 12).

Naylor and Nyanjom (2020) accurately highlight that because digital technology is constantly progressive it “tends to place educators in a position of perpetual novice” (p. 2). A recent report on the impact of moving to online learning and teaching during COVID-19 restrictions in Australia and New Zealand (Flack et al., 2020), also identify widely differing levels of confidence in various technologies being used for teaching remotely. The stress on internet servers with increased usage, as well as the capacity of household internet quotas to cope with all family members learning and working from home were additional concerns. Affordances were noted by respondents, but not as often as constraints. One respondent in Watermeyer and colleagues’ (2020) study felt that HE needed a catalyst like COVID-19 to challenge underlying assumptions about learning and teaching “my feeling is that the whole ethos of HE is likely to become more ‘modern’ and ‘innovative’ and as a result of these much more inclusive and accessible” (p. 15).

### Teacher and student well-being: an ethic of care

Teaching is inherently a social practice, and care is integral to successful teaching. Rose’s (2017) seminal position paper on social presence describes the ‘facelessness’ inherent to online teaching, especially with asynchronous delivery. These ‘faceless’ online environments have re-characterised HE as a form of “commodified, efficient, and disembodied information transfer” (p. 25). Rose, suggests therefore, that our decisions as educators should not merely be about enhancing how and what to teach by using “clever presence-enhancing techniques and strategies” (p. 23) but must be about overcoming the challenges to facilitating and maintaining caring relationships with students having a presence. Both Friesen’s (2011) and McShane’s (2006) pre-pandemic research concluded that this ethic of care is more difficult to develop online as the facilitator cannot adequately convey “empathy, trust, passion and emotion” (p. 202), and suggests, therefore, that teacher-student relationships are less genuine.

The vast majority of respondents in Watermeyer and colleagues (2020) research viewed the move to online teaching and learning as a negative experience, feeling that their pedagogical praxis had been reduced to “rudimentary technical functions” (p. 9). One respondent missed the personal connections in the classroom and described feeling like a ‘faceless’ purveyor of education, expecting their role was now to “transmit academic information rather than teach and model an academic ethos” (p. 10). What respondents did not predict was how much their pastoral role would increase, with more time spent counselling students and worrying about how to support those with mental health concerns. In addition, an ‘exponential’ increase in workload and demands on their time was highlighted leading respondents to feel exhausted and socially isolated.

The recent Pivot Report (Flack et al., 2020) detailed the three top concerns of 3,500 teachers from Australia and New Zealand across all school sectors during the move to online learning. The most common concerns were about students’ social isolation, a decrease in student well-being, and the loss of learning. Notably, this research suggests that educators rank students’ social needs above perceived learning loss and technological issues. Respondents expressed anxiety about a loss of social connection with their students and a decrease in the effectiveness of their teaching practice with one respondent stating, “not only do we teachers miss the social connection with our students, we miss being with our colleagues and friends…teaching is successful when connection is strong” (p. 4). Another felt that online teaching was a useful tool to support in-class teaching, but that the loss of subtleties of human interaction experienced in the same physical space could not be compensated for online. Respondents also pointed to the difficulty with the blurring of their professional and personal lives and responsibilities due to working from home, eroding any work-life balance that was established. Ultimately, it is the people skills, such as the skills of tolerance and cooperation, that Franklin (as cited in Freeman, 2014) cautioned are the most significant of what is lost through online learning and “the solitude of a computer screen” (p. 123).

## Methodology

The research team (Phillips et al. 2021) designed a qualitative survey which opened on May 4, 2020, when most countries were quickly moving to remote teaching online. The team consisted of 10 education researchers from HE institutions in Australia, New Zealand, Singapore, and the US, led by Associate Professor Louise Phillips from Southern Cross University. This online survey focused on how teachers and their students experienced the rapid change in learning environments during the first months of COVID-19 restrictions and was aimed and distributed to cross-sectoral teachers across the globe. Considering the central emphasis on teaching as a social, emotional, and personally connected profession, narrative identity theory was determined to be the most fitting methodology as it promotes “the constitution of identity through intersubjectivity” (Farquhar, 2012, p. 297). That is, an exploration of who we are in relation to others. In education, use of this methodology underscores the importance of the teacher as part of a social network and wider ecology of professional practice. Whilst the survey was left open for a longer period of time, for the purposes of this paper data was downloaded at the end of July, and as such the data collection period was from May 4, 2020, to July 27, 2020, which was a period of 84 days.

The participants’ evolving life stories (serve as personal reflections on the self-examination of professional identities as educators navigate the unknown and unpredictable. The respondents made narrative sense of their response to adversity, and how they imagine a future “with some degree of unity and purpose” (McAdams & McClean 2013, p. 233). Although agency and autonomy are integral to the teaching profession, suggests Farquhar (2012), the teacher works in relation to others and in this context, therefore, narrative identity theory prompts participants to ask, “who am I as an educator?” (Farquhar, 2012, p. 297). The researchers envisioned that not only would the data provide important lessons to inform future instances where similar restrictions may be put in place, but also contribute to the participants’ reimagining of their identities in a helpful manner, as previous research suggests that when narrators derive hopeful meaning from adversity in their lives “they tend to enjoy correspondingly higher levels of psychological well-being, generativity, and other indices of successful adaptation to life” (McAdams & McClean 2013, p. 236).

### Participants

Using a snowballing recruitment technique via social media, the participants in the study were 635 teachers working in early childhood education, primary schools, secondary schools, and university settings. This article reports on the 105 respondents in the HE/tertiary sector. There were 42 from the USA, 40 from Australia, with three each from Singapore and Canada, two from Japan and Fiji, and 13 other countries that had one respondent each. Each respondent was given a numeric code which was used in the analysis. The age range of the respondents was 40% (n = 42) aged between 45 and 54, 26% (n = 27) aged between 55 and 64, and 20% (n = 21) aged 65 and over. Only one person was aged 20–24, and three were 25–34. The next smallest age group was the 35–44 age group with 10% (n = 11) of respondents. In this HE subset 72% (n = 75) had either a PhD or EdD (Doctor of Education), whilst 22% (n = 23) had some sort of post graduate qualification and 6% (n = 6) had a degree. From this cohort 53% (n = 56) had over 21 years of teaching experience, whilst 21% (n = 22) had 16–20 years of teaching experience and 11% (N = 12) reported teaching for 11–15 years. This meant that 85% of our participants had over 10 years of teaching experience. Only 8% (n = 8) had 6–10 years’ experience and 6% (n = 6) had been teaching for 3–5 years and just one participant was in their first year of teaching. Interestingly, only 10% (n = 10) of the respondents had already been teaching online before remote emergency teaching began.

### Methods

This study was designed using survey-based methodology which allowed it to be based online using Qualtrics, an online survey tool to create the survey and host the survey. Using a snowballing technique, participants were able to pass the survey onto other teachers they knew who might be interested in completing. Social media was also used to allow for distribution of the survey as well as professional bodies in Australasia such as ASCILITE and HERDSA.

Prior to the research being conducted, the authors obtained human ethics approval through their respective institutions and co-designed a qualitative survey consisting of 16 open-ended questions with Qualtrics software used to gather responses (see Appendix A). The initial questions were devised to source demographic data such as the teachers’ age, discipline areas, years of experience in teaching, and previous experience teaching online. The next questions centred on their perceptions of how their students’ learning had been affected with a focus on adjustments for diverse learning needs. Questions then addressed technological, pedagogical, and personal needs, as well as challenges and support systems. Finally, participants were encouraged to share a story of success and reflect on what they had learnt about themselves as educators and co-learners. When using participant quotes to support the themes, each participant was given a number preceded by ‘Pt’ (e.g., Pt43).

## Data analysis

### Initial encounters and approach to analysis

With data collection beginning in May 2020, this study was opportune for participants and researchers alike. The respondents were particularly giving of their time, sharing challenging personal experiences and in more detail than expected. Our initial encounters with the data in digital spaces (Author, 2020) suggested that this may have been the first time the respondents took time to reflect on the ‘chaos’ that ensured when COVID-19 restrictions transformed their teaching instantaneously. Indeed, it appeared particularly cathartic for some. The importance of this opportunity was often noted: *We need each other, and sharing our stories brings us closer together* (Pt48)*.* These stories were captivating. Some made us smile and others caused us to shed a tear. As fellow HE teachers, we could empathise deeply. Through this emic perspective, it became obvious that we needed to engage with the data comprehensively, across themes and not confined by the parameters of each question, to gain a sense of the whole and the nuances that lay within. We chose to follow several complete narratives, building representative case studies to analyse. With more than two thousand lines of data, this took considerable time, but we appreciated the detail and wanted to honour this.

As an interdisciplinary research team, the data was interrogated by each researcher separately. The data was coded in Atlast.Ti (https://atlasti.com/) and the coded result exported to Excel to enable basic counts and listing of codes sorted by Respondent ID or by Code. Responses were assigned an ID number so that the coded results could also be sorted by Respondent ID or by Code. Coding followed a process of assigning a descriptive label or code to a comment or quotation; these text selections ranged from a phrase to several sentences. As higher education teachers, the researchers did not deny possible bias, but recognised and utilised the advantages of this emic perspective in the analysis.

When discussing emerging themes, it was apparent that responses aligned strongly with those of Kim and Asbury (2020). Their reflexive analysis which focused on teachers from primary and secondary schools, identified six themes around how teachers responded longitudinally to the abrupt impact of moving teaching and learning online. They suggest comparing research in other educational contexts to determine if similar themes emerge. Noting this call our analysis utilises their themes: *uncertainty, finding a way, worry for the vulnerable, importance of relationships, teacher identity, and reflections*. We have added subtitles to these themes to reflect our specific findings which were garnered through coding of themes from the data. This was completed by the team to ensure that the themes were systematically found, addressed, and reported. We also noted clear examples of and Naylor and Nyanjom’s (2020) teacher orientations.

## Results

### Theme 1: “Uncertainty”- technology, course redesign, and workload

As educators experiencing what we were researching (that is, unexpectedly moving to online teaching and learning), we had expectations of what we might encounter. Initial tales of frustration, exhaustion, and anxiety were prominent. This inceptive fear and confusion strongly aligned with Kim and Asbury’s (2020) theme of *uncertainty*.

Our participants, whilst seasoned and experienced educators were hit with the reality of hurriedly redesigning their lessons for online delivery. They spoke of a *quick race to develop online materials* (Pt40). They emphasised a sense of *confusion and some panic from students* (Pt40)*,* and that they had *difficulty answering questions about how the semester will proceed* (Pt40)*.* For educators that take pride in the quality of their materials and delivery, this sudden rush was *reactive rather than proactive* (Pt29). One respondent from the USA with over 21 years’ experience as a HE teacher explained this experience:

All courses were moved online with two-day notice. Not only had I very little experience with distance learning, but also little interest. Fortunately, I had had seven weeks with my students [prior to closures] so that the relationships were established, but I had a steep learning curve to make the switch to online. The chief impact was fear of a platform I dreaded and losing treasured rituals in the classroom (Pt56).

This disorientation caused by a sudden change in teaching routine is what Naylor and Nyanjom (2020) have identified as a main cause of emotional responses from teachers. Respondents who felt disempowered and vulnerable by the move to online instruction (Downing & Dyment, 2013) would appear to be ‘cautious’ educators. Their concerns were about a medium of instruction perceived to be passive, through which they would struggle to motivate and engage students, as they were confident and adept in doing so in the same physical space and using their well-developed routines.

As one might have expected, there were core concerns such as learning about unfamiliar technologies and changes to teaching styles and resources. For some, the transition was not too onerous: *Online teaching is the norm at my university. I am used to it and familiar with the tools needed to conduct such teaching* (Pt14), but as only 10% of respondents had taught online previously, the majority needed to *quickly adapt teaching styles, lesson plans, and assessment tasks to suit the online environment* (Pt14). They dealt with problems such as poor internet connections, trouble-shooting students’ technical issues, and slow transitions between activities. The Pivot Report (Flack et al., 2020) notes similar findings, in which the need to engage competently with a range with digital tools left some teachers on the back foot and in the ‘role of a perpetual novice’ as they negotiated regular updates to programmes and applications (Flack et al., 2020). When anxiety is high, notes Trigwell (2012), teachers are regrettably more likely to adopt ‘safe’ and teacher-centred approaches, regressing from any gains made towards student-centred learning.

Experienced teachers may know how to command the physical classroom, but many of our respondents expressed that *it’s harder to read the ‘room’ when many students don't show their face and harder to tell if students are understanding or responding to the work* (Pt31). Where learning was typically student-focused this change of roles was stressful: *[Online] tutorials are weird. When in-person, I—as the tutor/facilitator—generally want to initiate a discussion and then watch the students talk it out. Online it is much more ‘me-focused’. That’s exhausting, and I don't like it* (Pt39). Olivera et al. (2021) reported similar frustrations in their study of HE teachers and students in Portuguese speaking countries. Most of the time students had their cameras turned off and as such, teachers could not ascertain if they understood the concepts or skills being taught. One student offered “In many of the moments I looked at the screen, I didn't understand anything” (p. 1370) confirming this disjunct.

Bigger concerns evolved with engaging students, especially when they had been online since 8:00am and were losing focus. *Students are experiencing screen fatigue, and this makes their motivation levels hard to sustain*. *I have to work much harder for engagement* (Pt35)*.* For teachers whose ways of working typically rely heavily on praxis, this move to online delivery was especially challenging: *I am really struggling with how to have the interactive parts of my courses remain as meaningful as they are when we are face-to-face* (Pt88)*.* One Drama teacher from Australia with over 21 years’ experience, experimented with a variety of ways to approximate Drama praxis, but without success: *Students have appreciated the efforts we went to continue their Drama education but have hated it. They felt disconnected and terribly unmotivated* (Pt22)*.* The creative arts are almost exclusively experienced as praxis, “physical, embodied learning that utilises all senses” (Author 2021b under review). Davis and Phillips (2021) report that Drama teachers missed the “embodied, social and relational aspects of learning” (which are promoted through Drama pedagogy) when learning moved online (p. 2). This can be compared to the reciprocity of meaning-making that Platisdou (2010) refers to, and which boosts a sense of community and connection.

The ultimate price for dealing with this uncertainty was ‘losing’ students in the learning process: *Some students told me right away that online classes were too difficult, and it was not the way they learn* (Pt82)*.* And for a minority of teachers, regrettably, it was the catalyst for ending their careers:

I tried to teach online as I’d always taught and it just didn't work. It was frustrating for the students and demoralizing for me. I'm a good teacher and still have much to share with young people. I sadly and reluctantly resigned at the end of the semester because I found my online teaching to be artificial, ineffective, and very unsatisfying. (Pt57)

Reponses in this theme demonstrate the stress and anxiety felt by teachers as they quickly moved into an unknown space. As Kim and Asbury (2020) suggest, this uncertainty and unpredictability is akin to leaping out of a plane with only a diagram of how the parachute works. For many, navigating new technological knowledge and leaving behind trusted ways of interacting with their students lead to disorientation and frustration.

### Theme 2 “Finding a Way”—making adjustments and moving forward

When teachers realised that the restrictions were not short term and that it was possible online learning would continue indefinitely, they reassessed their positions and pushed on to meet their students’ needs. Making necessary pedagogical adjustments and preserving teacher well-being fits comfortably with Kim and Asbury’s (2020) theme of *finding a way.*

In *finding a way* many respondents noted a significant increase in workload: *I’ve been working 7 days a week for months now with the odd weekend day off. And no end in sight* (Pt34)*.* And yet, not one respondent mentioned limiting their workload to working hours. Teachers with children at home felt added stress: *The lack of physical separation from a workplace makes it hard to ‘turn off’. And trying to help with school while my children were home at the same time as working was a huge challenge* (Pt39)*.* Respondents knew that continuing to be available ‘24/7’ would result in jeopardising their physical and mental health: *I am going to have to be mindful to have better boundaries so I don't burn out* (Pt88)*.* One teacher described being challenged mentally, physically, and practically:

Mentally I am more likely to become frustrated, and even angry with colleagues, students, and administrators. I've discovered I'm not a great person to have or be around in a pandemic. Physically I am sitting far too much. I have developed a ‘zoom face’ which is a squinty-frown as I try to work out who is talking and why they're still in bed. (Pt26)

Taking time for walking, working in the garden, measured breaks, hobbies, prayer, connecting with friends and loved ones, and maintaining a healthy diet were mentioned as ways to release stress. *I need breaks to regroup. I need to step away from my computer and intentionally move or I get too focused and forget to eat and exercise* (Pt48)*.* Our review of current research shows a growing concern for students’ mental health, well-being, and socio-emotional needs, but there are limited studies on the impact of COVID-19 restrictions on teachers. Casacchia et al.’s (2021) research with HE teachers in Italy noted impacts similar to those reported by our respondents. These include increased appetite, exhaustion, anxiety, and stress resulting from teaching online, which they term this ‘technostress’. In addition, there were impaired sleep patterns (in more than 50% of respondents) and a loss of energy which invaded the personal life of teachers, which they termed ‘technological invasion’.

Almost exclusively, teachers let us know about the extraordinary lengths they went to support their students. This included being available outside class hours for one-on-one sessions online or on the phone: *Adding in many extra contact hours so they feel they are getting enough individual support* (Pt35)*.* Changes in teaching strategies included breaking up longer seminars with more frequent shorter ones, staying online after sessions for those who have questions or want to talk more, offering drop in Q&A sessions, providing more comprehensive feedback on assessments, extending due dates, providing more overt demonstrations of empathy, and regular checks on students’ emotional health were trialled: *There was always a well-being check in at the beginning of class and some discussion as to how COVID-19 was impacting students* (Pt12)*.*

Although online delivery is not the mode that most respondents would have chosen, there was a realisation that it came with some positives: *It made me seek alternative ways to deliver teaching and learning, to become more resourceful especially with accessing online resources* (Pt96)*.* And that some teaching strategies were, in fact, best suited to the online environment: *Some insightful discussions have been had and we were able to invite global experts to participate in lessons. This would not have been otherwise possible (Pt96).* Respondents noted skills they did not know they had or probably would not have developed if the COVID restrictions had not come into play: *I am capable of managing a level of technology that I wouldn’t have chosen to do or believed that I could do* (Pt92)*.*

This theme of *finding a way* saw teachers realising they needed to move forward by building skills and knowledge. Still impacted by the initial uncertainty, they found ways to deliver quality education with what skills they had. Teachers understood that to manage the increased workload, they needed to practice self-care. A realisation that they were capable of more than they first thought buoyed their progress.

### Theme 3 “Worry for the vulnerable” – the haves and the have nots

New rituals and ways of connecting with students brought on new concerns for how they were coping emotionally and financially: *For most of [my students], the distractions were so great and the emotional toll so high…there is a general sense of chaos and anxiety that make concentration difficult* (Pt76). These concerns were pervasive, as noted similarly in Kim and Asbury’s (2020) theme of *worry for the vulnerable.* The move to online instruction served to highlight the deep inequities that currently exist, especially for students with disability, students for whom English is an Additional Language or Dialect, and students from low socioeconomic contexts. The impact of such factors on learning were intensified at this time: *The lived realities of teaching have deepened my understandings of trauma-informed teaching, particularly with underrepresented students* (Pt81).

The change was less of a burden for students who had what they needed; both technology and support: *Students who have internet access, materials, and do not have outside responsibilities seem to be able to engage with learning* (Pt81)*.* A teacher in the USA, however, knew her cohort of students would be disadvantaged due to the societal impacts of COVID-19:

My institution serves many minority, underprivileged, and non-traditional students. Due to COVID-19, many students had to pick up extra shifts. One of my students, who works in a dollar store, told me that she worked more to fill in the gaps left behind by her colleagues who fell ill or had to provide care for children after schools closed. Another student had to pick up as many shifts as she could at her job so that she could support her family after both of her parents were laid off. Other students did not have computer and internet access at home. (Pt83)

Teachers purposefully switched their priorities from content delivery and assessment, to care and compassion: *Kindness, caring, and empathy are key* (Pt83). They realised that some students were truly scared and needed support: *This spring semester I made it very clear that the students’ health and their families’ health was #1. Anything else was secondary* (Pt78). As Rawle (2021) recently shared, a pedagogy of kindness is the cornerstone for students learning and wellness during this pandemic. Whilst some problems could be solved, HE teachers were not always able to assist their students, particularly those with little or no internet access: *My students are often poor and have difficulty accessing computer equipment and reliable wifi. Many of my students are First Nations and after the pandemic was declared, they moved back to their home reserves, which often do not have wifi* (Pt88)*.* Also, for those in Pacific Island counties, restrictions made getting to campus to use computers insurmountable: *Students cannot get to the campuses especially when they are in remote areas. Travel by bus or boat has been prohibited. Even here in the main campus, movement is restricted* (Pt90)*.* This concern for well-being was not one way. Several respondents recognised that their students now saw them differently: *Some have realised that professors are human too* (Pt76). They witnessed that *students have become emotional, more empathetic, and more humane* (Pt76) (Cañas-Lerma et al., 2021).

The responses in this theme demonstrated that it was not long before teachers realised how remote learning illuminated existing socioeconomic inequalities. Their concern for the most vulnerable- medically, academically, and emotionally, was a clear focus.

### Theme 4 “Importance of relationships”—missing the connections

There was no theme more prominently explored than that of personal connections between teachers and their students. Respondents’ stories resonated profoundly with us as we were experiencing the same yearning to be back in the classroom. By moving learning online, teachers felt they had lost the opportunity for deep spontaneous discussion and rapport building. *I miss the interaction with students. I am less motivated and enjoy my teaching less. I have become a curator and creator of learning resources rather than an educator* (Pt33)*.* Teachers realised that they treasured face-to-face interaction which privileges body language and nonverbal cues. *I believe that teaching is about relationships, and it is far more difficult to establish relationships in an online environment* (Pt56)*.* This visceral reaction to the loss of personal connections and changes to relationships is echoed in Casacchia and colleagues’ (2021) study with HE teachers in Italy. Documenting the technical, instructive, and psychological challenges experienced, by far the most distressing impact was the feeling of speaking into the abyss of a computer screen. Their respondents acknowledged that working with students in the same physical space energised instruction and that the act of learning was far more than the transmission of knowledge, but also “emotions, feelings, relationships, and positive memories” (p. 16).

Students also missed face-to-face interaction: *They tell me that they miss the personal relationships and rich conversation that goes on in classrooms the most. Many are worried about speaking out online* (Pt40)*.* Our respondents drew links between the loss of emotional connection with students and their lack of academic growth. Knowing this, prompted some to find creative ways to reach out to their students. Relationship building became the priority with an emphasis on the class understanding each other as individuals: *Sending a survey to students prior to the first class that draws on their unique skills and abilities and getting them to talk, bond and get to know each other prior to teaching any content. The first class is the students sharing who they are, what they want to do, their goals, dreams, passions and what they want to get out of the class* (Pt18)*.* Teachers with high-perceived emotional intelligence (the ability to accurately receive and regulate emotions) suggests Platsidou (2010) are likely to feel less emotional exhaustion, have lesser rates of burnout, and have a high sense of personal accomplishment. We suggest therefore, that higher levels of emotional intelligence may be important in weathering periods of online learning where emotional connections are harder to maintain.

Teaching is a fundamentally social endeavour. It is about people and relationships. Through COVID-19 restrictions, educational relationships changed with teachers finding new and creative ways to build and maintain a connection to their students. Teachers cared if their students were progressing. Teaching online did nothing to dampen their responsibilities as educators.

### Themes 5 and 6: reflections and teacher identity—‘Who am I as a teacher?’

After our respondents grappled with the most pertinent issues and concerns, they emerged from the confusion and panic, took a breath, and surveyed the result. For some this meant looking at the big picture and asking, “who am I as an educator?” We see this as aligning well with Kim and Asbury’s (2020) themes of *Reflections* and *Teacher Identity.*

For many, their identities as teachers and well-honed pedagogical styles were tested and challenged: *This has been a period of growth as I have had to learn how to teach differently and learn new skills* (Pt37). A literacy teacher from the USA made this assessment: *Teaching through an online interface has encouraged me to deeply examine my underlying theoretical understandings regarding teaching and learning. I teach through a relational lens, and so have explored ways to build relationships with students in online environment* (Pt41)*.* What stood out was a pedagogy of care and kindness for both their student and themselves: *We are all in this together. Chaos is assumed and we are doing our best.* Despite their shortcomings, respondents acknowledged that were still making a positive impact: *Even if what I am doing is not perfect, I hope the students can see that I actually care about them and their learning. Their little emails of thanks and acknowledgement are very sustaining.*

There were many evaluative statements found in the data that began with ‘I’. This personalisation, sense of autonomy and agency, and trust in the study demonstrated that the participants were learning about themselves, moving forward with their new identities in these times of crisis and wanting to share this shift. ‘I’ statements included:

I have learned that I can adapt and overcome under extreme pressure (Pt44);

I can make the best of things and learn regardless of the obstacles (Pt56);

I learnt that planning to teach online has improved my in-person teaching (Pt59);

I am flexible and patient in times of stress (Pt60);

I thrive on relationships in person (Pt40);

I care deeply about my students’ abilities to manage online (Pt69);

I am more focused on the emotional and sociocultural aspects of learning (Pt73);

I have learned that teaching online isn't that scary but that I really do miss the face-to-face interaction (Pt20);

I really care about my students, and there are many ways to reach students (Pt30).

These storied reflections demonstrate that HE teachers hold onto what is familiar and personally important in times of stress, but proactively employ problem-focused strategies such as learning new skills and redeveloping their course material. These included emotion-focused strategies such as seeking emotional support and preferencing the effect of newly acquired pandemic pedagogies and relationships over content (Kim & Asbury, 2020).

## Conclusions and implications for teaching practice and educational research

McLean and colleagues suggest that “stories are the substance of the self” (2007, p. 275). We were privileged through this research to be the recipients of data stories about public and private selves, which integrate to develop narrative identity of professional selves during the early stages of the pandemic and pivot online. Storytelling opens a path for others to follow in, see themselves and their experience with, and offers space to feel the effect of the encounters we find ourselves in during the pandemic. As HE continues to shift post-pandemic, capturing the stories and the feltness of emerging pandemic pedagogies in this study has been important, because, as Kimmerer (2013) suggests, “stories are both history and prophecy, stories for a time yet to come” (2013, p. 207). This highlights the importance of capturing the visceral embodiment of stories for now to serve as learning for future generations.

Returning to the research question ‘*in what ways did the immediacy of the shift to online learning during COVID-19 times impact teachers’ identities*?’ we see that our respondents’ identities as teachers were impacted in multiple ways. After the initial tellings of confusion, frustration, exhaustion, vulnerability, and anxiety, the narratives refreshingly and perhaps unexpectedly moved purposefully towards understanding, meaning-making, agency, creativity, and hope, with a central emphasis on relationships and a pedagogy of care and kindness in the online space. The depth that respondents provided us in their stories demonstrates that narrative builds over time and as people share their experiences with others. Over time, suggests McLean et al., (2007) in their sociocultural model of narrative identity, *selves create stories which in turn create selves*. Whilst our respondents found the sudden shift to remote online learning challenging and stressful for many reasons, as they encountered many technological and pedagogical obstacles many of our participants told us about their agility to remain flexible, adaptable, and resilient throughout the challenges of online delivery in their situated stories. McLean et al., (2007) use the term ‘situated stories’ to emphasise the fact that “any narrative account of personal memory is created within a specific situation, by particular individuals, for particular audiences, and to fulfill particular goals” (p. 262). This storying of their situations allowed our participants to reimagine their identities as teachers and co-learners. Research by McAdams and McClean (2013) suggests that when narrators derive hopeful meaning from adversity in their lives “they tend to enjoy correspondingly higher levels of psychological well-being, generativity, and other indices of successful adaptation to life” (p. 236). Learning through adversity has seen our participants come out the other side as more skilled, empathetic, and creative teachers. Should they be forced to teach online in the future, they will be ready to meet that challenge.

The number of participants in this study is healthy for a qualitative study, however, limitations are evident. A wider sample from higher education teachers in developing nations, countries with a large geographical area, and countries limited resources, remoteness, and susceptibility to natural disasters such as Small Island Developing States would provide a more nuanced understanding of how students without access to internet and technology devices are affected when they are not able to come to campus.

We have often heard COVID-19 referred to as a ‘once in a lifetime’ pandemic that has brought about a unique teaching and learning landscape. But the reality is that we will face ongoing disruptions to learning from a whole range of sources. Fires, flooding, and other natural disasters will strike, necessitating teachers to once again pivot to remote learning. As such, there are significant findings and wide-ranging implications from this research in that this tells the story of HE teachers caring for students, of worrying about mental health challenges and how best to support learners and learning during a health crisis. It not only archives a developing understanding of agile, nimble, and caring pandemic pedagogies in HE. It tells a new story of responsive and relational education in a health crisis through vibrant reflective moments for storytelling and belonging in an online community through this research. As a result, there are implications for teaching teams, teaching practices and pedagogies, research teams and communities of practice across HE.

## Survey questions

1. How has COVID-19 impacted your teaching and learning?
2. What is different about your delivery?
3. How has the children’s/students learning been affected?
4. How are you addressing diverse learning needs and approaches, cultural relevance and cultural responsiveness in the altered practices to teaching and learning?
5. What are your students’ questions and concerns and how do you address them?
6. What are the issues you are struggling with and need support with?
7. What online platform/s are you using?
8. What have you changed with regard to your teaching to support students?
9. What are the strategies that your students are using to study online?
10. Who and what are your key knowledge sources for teaching remotely?
11. What new partnerships have you formed to deliver teaching and learning?
12. What innovations have you forged or experimented with?
13. Please share a story of a successful teaching and learning encounter.
14. What have you learnt about yourself and your teaching?
15. What helps you get through each day?
16. What do you think your students have learnt broadly about these changes (such as about humanity, about themselves as learners)?
